# Relationship between triglyceride–glucose index and carotid plaques in a high-stroke-risk population in southeast china: A population-based cross-sectional survey

**DOI:** 10.3389/fendo.2022.1023867

**Published:** 2022-10-12

**Authors:** Xiang Tang, Lulu Zhang, Yidan Li, Yun Zhou, Xiuying Cai, Ye Yao, Qi Fang

**Affiliations:** ^1^ Department of Neurology, First Affiliated Hospital of Soochow University, Suzhou, China; ^2^ Department of Biostatistics, School of Public Health, Fudan University, China and National Clinical Research Center for Aging and Medicine, Huashan Hospital, Fudan University, Shanghai, China

**Keywords:** triglyceride–glucose index, cervical arterial atherosclerosis, carotid plaque, stroke screening, insulin resistance

## Abstract

**Background:**

Cervical arterial atherosclerosis (CAA) is an important risk factor of stroke in China. The triglyceride–glucose (TyG) index is a simple and low-cost marker for ischemic stroke. Whether the TyG index predicts cervical arterial atherosclerosis remains uncertain. This study aimed to investigate the relationship between the TyG index and cervical arterial atherosclerosis.

**Methods:**

This cross-sectional study was conducted in residents aged ≥40 years in the general population of southeast China. All participants completed a detailed questionnaire and provided blood samples. The high-stroke-risk groups further completed cervical artery ultrasonography. The TyG index was calculated using a well-established formula and analyzed in quartiles (Q1–Q4). Multivariate logistic regression was used to investigate the relationship between the TyG index and cervical arterial atherosclerosis.

**Results:**

A total of 4,499 participants aged ≥40 years were finally included, with 23.47% comprising the high-stroke-risk population. The prevalence rates of increased intima–media thickness (IMT), carotid plaque, and cervical artery stenosis (CAS) in the high-stroke-risk population were 21.97%, 39.3%, and 6.1%, respectively. Subjects with higher TyG were still more likely to have carotid plaque. After adjusting for several established risk factors, compared with the TyG-Q1 group, the TyG-Q2, TyG-Q3, and TyG-Q4 groups were more likely to have carotid plaque (OR = 1.85, 95%CI = 1.28–2.67; OR = 1.51, 95%CI = 1.05–2.18; and OR = 1.29, 95%CI = 0.90–1.84). TyG was an independent predictor of the presence of plaque in the carotid artery of the high-stroke-risk population.

**Conclusions:**

An elevated TyG index is a potential predictor of carotid plaques in the high-stroke-risk population older than 40 years.

## Introduction

Stroke ranks as the third cause of death and disability in China, which has been a threat to human health (1). China has the highest estimated lifetime risk of stroke, which increases the total cost of stroke. Reducing the incidence of stroke ensures quality of life for adults and has a positive impact on individuals, families, and the society. Stroke is not an accident. Stroke screening for the early detection of a high-risk subject and managing the risk factors are of great significance to the prevention of stroke. Primary stroke prevention can reduce the incidence of stroke and lessen its financial burden (2, 3).

There are numerous causes of stroke, including prolonged hypertension, arteriosclerosis, and emboli (4). Atherosclerosis is a chronic condition that causes an accumulation of fatty streaks in arterial walls, which develop into atheromas and plaque. One-third of all strokes are related to cervical carotid disease. The risk factors for coronary and systemic atherosclerosis, including age, sex, hypertension, hyperlipidemia, unhealthy lifestyle habit, and family history, apply to this patient population. Cervical arterial atherosclerosis includes increased intima–media thickness (IMT), carotid plaque, and cervical artery stenosis (CAS). The mechanism of cervical carotid stroke is usually embolization from the carotid bifurcation plaque and/or hemodynamic compromise from stenosis (5). Ultrasonography is a good method for detecting ceramic arterial atherosclerosis. However, due to the requirement of professional equipment, the need for experienced ultrasound physicians, and the higher cost of ultrasonography, it is impossible for the general population of grassroots hospitals in villages and towns to complete ultrasound examinations.

The triglyceride–glucose (TyG) index is a product of triglyceride and fasting blood glucose (FBG) and has several related parameters, such as the product of TyG and waist circumference (TyG-WC), TyG and waist-to height ratio (TyG-WHtR), TyG and waist-to-hip ratio (TyGWHpR), and TyG and body mass index (TyG-BMI) (6). The measurement of TyG is inexpensive and easily obtained, and it is suitable for use as a screening indicator for the general population. The TyG index is a novel surrogate indicator of insulin resistance (IR). Therefore, initial studies have suggested the TyG index as a novel marker for multiple IR-related clinical diseases, such as metabolic syndrome (MS) (6). It provides good discrimination of people with prediabetes and diabetes (7). In recent years, the TyG index has been studied as a novel tool for evaluating atherosclerosis of cardio-cerebrovascular diseases (CVDs) in different populations. This index may serve as a marker for subclinical atherosclerosis and arterial stiffness in lean and overweight postmenopausal women (8). Researchers found that the TyG index could predict the IMT of the common carotid artery (CCA) in hypertensive individuals, which is an important risk factor for stroke (9). Jin et al. found the TyG index to be positively associated with future cardiovascular events and may be a useful marker for predicting clinical outcomes in patients with coronary artery disease (CAD) (10, 11).

Previous findings also suggested the potential value of TyG and the TyG-related parameters in optimizing the risk stratification of ischemic stroke. In a cross-sectional study that included 10,900 subjects from rural areas of northeast China, the prevalent ischemic stroke correlated proportionally with the increment of TyG, implicating the linearity of TyG as an indicator of ischemic stroke (12). In another rural Chinese cohort study, an elevated TyG also predicted the risk of incident ischemic stroke (13). Sun et al. found a robust correlation between TyG-BMI and ischemic stroke, independently of a host of conventional risk factors in populations of northeast China (14). In addition to this, the TyG index can predict functional outcomes and mortality after acute ischemic stroke (15–17). It is also a potential predictor of hospital and intensive care unit (ICU) mortality in critically ill stroke patients, especially in ischemic stroke patients (15). On the other hand, Hou et al. suggested that the TyG index may not be beneficial in understating the metabolic mechanisms responsible for the stroke obesity paradox (18). In summary, the correlations between the TyG index and stroke are inconsistent. To date, only a few studies have focused on the TyG index and cervical arterial atherosclerosis. The causal link between the TyG index and atherosclerosis remains unclear. Moreover, the most relevant studies in China have focused on the population of the northeast area.

Therefore, we aimed to investigate the associations between the TyG index and the occurrence of cervical arterial atherosclerosis in the general population of eastern China. Our hypothesis was that the TyG index is associated with and may be a predictor of cervical arterial atherosclerosis. Assessment of this relationship will help verify that TyG, a more convenient and low-cost index, has potential value in improving the risk stratification of stroke. For this reason, the present study examined the TyG index in different risk populations of eastern China based on the Stroke Screening and Prevention Program. On the other hand, the study further explored the association between the TyG index and carotid ultrasound indices in the high-stroke-risk population.

## Methods

### Study design and participants

**Figure 1:**
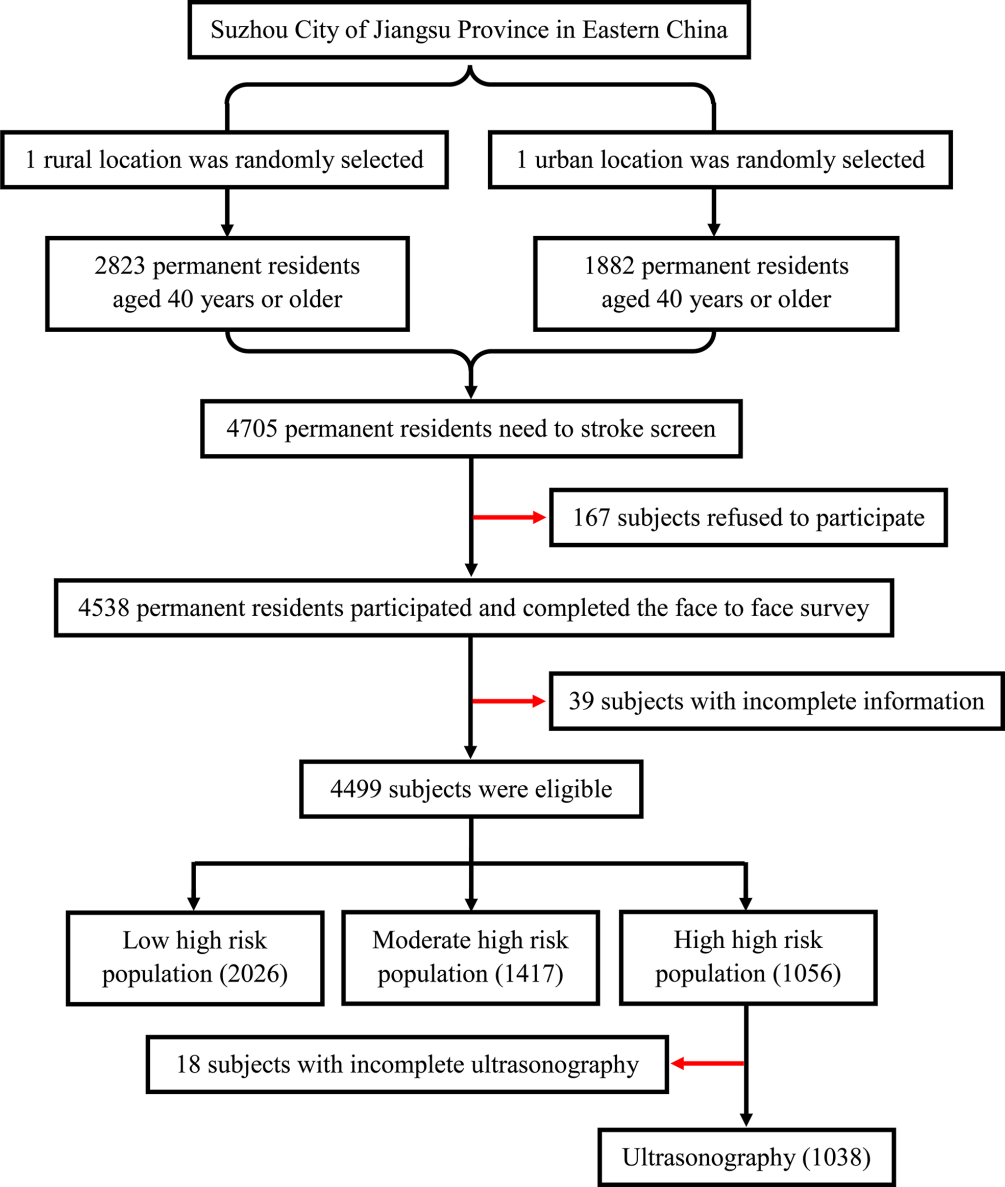
Flowchart of the enrollment of subjects.

The study population was from the Stroke Screening and Prevention Program of the National Health and Family Planning Commission of China, which was supervised by the Chinese National Center for Stroke Care Quality Control and Management (Stroke Prevention Project, National Health Commission). The project implemented stroke screening for urban and rural residents in Suzhou, Jiangsu Province, China. As a representative city, Suzhou is located in southeast China, southeast Jiangsu, and in the middle of the Yangtze River Delta. One urban and one rural location were selected randomly in Suzhou. The study was conducted from December 2018 to June 2019. The number of the final screening subjects accounts for more than 85% of the residents in this location. Finally, a total of 4,705 permanent residents aged ≥40 years who had lived in Suzhou City for more than 6 months were randomly selected, of which 4,499 individuals successfully completed the face-to-face survey. The sample selection framework is presented in **Figure 1**. This study was conducted according to the guidelines of the Helsinki Declaration. Ethical approval was obtained from the Ethics Committee of the First Affiliated Hospital of Soochow University before the start of the study. Written informed consent was obtained from all the participants.

### Data collection and laboratory Analysis

Data were collected during face-to-face interviews at a central survey site in the area. The participants completed a structured, pre-coded questionnaire including demographic details, personal lifestyle, and personal and family medical history of stroke and chronic diseases. Anthropometric measurements were taken following standardized protocols. Blood pressure was measured twice and the average taken by trained professionals. Venous blood samples were collected from an antecubital vein in the morning after an overnight fast for laboratory examinations. These indices included FBG, hemoglobin A_1C_, total cholesterol (TC), triglyceride (TG), low-density lipoprotein cholesterol (LDL-C), high-density lipoprotein cholesterol (HDL-C), and homocysteine (Hcy). All data were double entered and validated.

### Definition of terms and groups

The TyG index and related parameters were calculated as follows (6):

1) TyG index=Ln[TG (mg/dl)×fasting blood glucose (mg/dl)/2]
2) TyG and body mass index: TyG-BMI=TyG index×BMI
3) TyG and waist circumference: TyG-WC=TyG index×WC
4) TyG and waist-to height ratio: TyG-WHtR=TyG index×WHtR

The visceral adiposity index (VAI) was calculated using an established formula (19).

In men: VAI=(WC/(39.68+(1.88×BMI))×(TG/1.03)×(1.31/HDL)

In women: VAI=(WC/(36.58+(1.89×BMI))×(TG/0.81)×(1.51/HDL)

Low-, medium-, and high-risk populations of stroke were defined according to the “8+2” Stroke Risk Screening Program: 2 main risk factors and 8 general risk factors for stroke (20).

Eight general risk factors were described, as follows:

1) Hypertension: defined as a history of high blood pressure (≥140/90 mmHg) or current use of antihypertensive medication;
2) Atrial fibrillation (AF) or heart valve diseases: either reported by the respondent or defined as an irregular pulse during physical examination;
3) Smoking: defined as current or former practice of smoking ≥6 months;
4) Dyslipidemia: defined as current use of anti-lipidemic medication, TC≥6.22 mmol/L, or serum TG≥2.26mmol/L, or HDLC<1.04mmol/L;
5) Diabetes mellitus: defined as a previous diagnosis, treatment with insulin or oral hypoglycemic medications, fasting plasma glucose ≥126 mg/dl, or glycosylated hemoglobin≥6.5%;
6) Physical inactivity: defined as physical exercise less than three times a week for<30 min each (moderate-intensity exercise such as brisk walking; industrial and agricultural labor was considered as a form of exercise);
7) Overweight or obesity: defined as BMI ≥24 kg/m^2^; and
8) Family history of stroke

The additional two main stroke risks were:

1) Personal history of stroke and
2) Personal history of transient ischemic attack (TIA)

Subjects with at least three of these risk factors or a previous history of stroke or TIA were classified into the high-risk population. Subjects with up to three of these risk factors or with hypertension or diabetes mellitus (DM) or AF or heart valve diseases were classified into the medium-risk population. Subjects with three or less of these risk factors without hypertension or DM or AF or heart valve diseases were classified into the low-risk population.

### Ultrasonography

The high-risk groups further underwent cervical artery ultrasonography (MyLabSix, Esaote, Italy) examinations by experienced ultrasound physicians with at least 5 years of ultrasound experience. The bilateral CCA, internal carotid artery (ICA), the subclavian artery, and the vertebral artery were examined and recorded. IMT was measured manually three times in a plaque-free area of each CCA and the averaged thickness recorded. Both common carotid arteries were examined, and an increased IMT was defined as IMT ≥1.0 mm in either the left or the right carotid artery. Carotid plaque was defined as IMT ≥1.5 mm or a focal narrowing of the vessel wall of >50% relative to adjacent segments. The incidences of plaque morphology, ulcer, and echo were recorded. CAS included intracranial vascular stenosis and occlusion (19).

### Statistical analysis

Statistical analysis was completed using SPSS 22.0 (International Business Machine, West 31 Grove, PA, USA). Continuous variables were presented as the mean ± SD. Categorical variables were presented as frequency (percentage). Differences between groups were tested using one-way ANOVA with *post-hoc* analysis (Bonferroni) for continuous variables and chi-square test, corrected chi-square test, or Fisher’s exact probability method for categorical variables. Baseline characteristics of the high-risk groups were sorted by TyG quartiles. Multivariate logistic regressions were conducted to explore the relationship between carotid plaque and all risk factors. A chi-square goodness-of-fit test was applied and represented the goodness of fit in the logistic regression. We mainly included age, sex, and the “8+2” stroke risk factors in the final logistic regression models. Furthermore, the correlations between TyG and carotid plaque were calculated using logistic regression analysis in different models: model 2 was adjusted for age and sex; model 3 was adjusted for age, sex, smoking, drinking, and physical inactivity; model 4 was adjusted for age, sex, smoking, drinking, physical inactivity, hypertension, DM, heart disease and dyslipidemia, personal history of stroke/TIA, and family history of stroke; and model 5 was adjusted for the predictors that showed significant differences in the univariate analysis. We also performed several subgroup analyses for men, women, DM, non-DM, overweight or obese, non-overweight or obese, sweet tooth, non-sweet tooth, physical inactivity, and non-physical inactivity. All of the subgroup analyses were adjusted using the same parameters as described in model 4 in the main analysis. Receiver operating characteristic (ROC) analysis was performed to construct the prediction of cervical artery atherosclerosis. All reported *p*-values were two-sided, and a *p*< 0.05 was considered as statistically significant.

## Results

### Demographics and baseline characteristics of all participants

**Figure 2:**
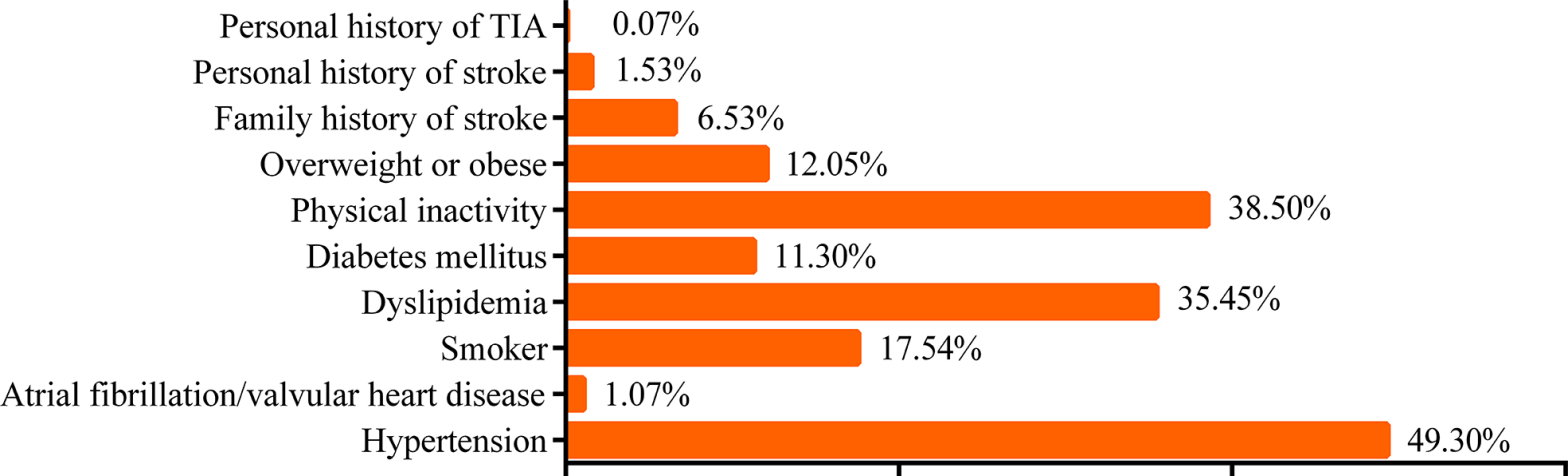
Risk factors according to the “8+2” Stroke Risk Screening Program. *TIA*, transient ischemic attack.

In total, 4,499 participants in the stroke screening (1,556 men and 2,943 women; mean age, 57.88 ± 9.79 years) were included in the analysis. Nearly 61.52% of the participants were from townships and 38.48% from communities. The smoking rate of the participants in this study was 17.54%. Among the included participants, 38.50% reported physical inactivity, 12.05% reported being overweight or obese, 49.3% had hypertension, 11.3% had DM, 1.07% had AF and/or valvular heart disease (VHD), and 35.45% had dyslipidemia. In addition, 6.53% of the participants had a family history of stroke. Regarding personal medical history, 1.53% had a personal history of stroke and 0.07% had a history of TIA (**Figure 2**).

According to the aforementioned diagnostic criteria of the “8+2” Stroke Risk Screening Program, the target population of stroke screening was divided into three groups: low-, medium-, and high-risk groups. All the demographic and baseline characteristics were sorted based on the risk of stroke and are shown in **Table 1**. The results showed the percentages of the three groups as 45.03%, 31.50%, and 23.47%. Of the participants, 61.75% have a sweet tooth. There were significant differences between the groups regarding family history of hypertension, diabetes, and coronary heart disease. The high-risk group was more likely to have a higher systolic blood pressure (SBP) (*p*< 0.001), weight (*p* = 0.002), waist circumference (*p*< 0.001), and BMI (*p*< 0.001). Concerning laboratory features, the high-risk group was more likely to have a higher VAI (*p*< 0.001), FBG (*p*< 0.001), hemoglobin A1c (*p*< 0.001), TG (*p*< 0.001), TC (*p*< 0.001), LDL-C (*p* = 0.008), and homocysteine (*p*< 0.001). In addition, the high-risk group had significantly higher TyG and values of the TyG parameters TyG-BMI, TyG-WC, and TyG-WHtR (all *p*< 0.001).

**Table 1:** Baseline characteristics of all participants.

	Total	Low-risk population	Medium-risk population	High-risk population	*p*-value
*N*	4,499	2,026 (45.03%)	1,417 (31.50%)	1,056 (23.47%)	
Region					0.46
Urban, *n* (%)	2,768 (61.52)	1,237 (61.06)	864 (60.97)	667 (63.16)	
Rural, *n* (%)	1,731 (38.48)	789 (38.94)	553 (39.03)	389 (36.84)	
Age (years)	57.88 ± 9.79	57.52 ± 9.87	57.84 ± 9.63	58.62 ± 9.84	0.01*
Gender, male, *n* (%)	1,556 (34.59)	798 (39.39)	404 (28.51)	354 (33.52)	<0.001***
Eating habits					<0.001***
High-salt diet	595 (13.23)	265 (13.08)	181 (12.77)	149 (14.11)	
Oil-heavy diet	1,126 (25.03)	428 (21.13)	383 (27.03)	315 (29.83)	
Sweet tooth	2,778 (61.75)	1333 (65.79)	853 (60.20)	592 (56.06)	
Meats and vegetables					<0.001***
Balanced	321 (7.13)	126 (6.22)	105 (7.41)	90 (8.52)	
Carnivorous diet	895 (19.90)	350 (17.28)	302 (21.31)	243 (23.01)	
Plant-based diet	3,283 (72.97)	1,550 (76.50)	1,010 (71.28)	723 (68.47)	
Daily vegetable intake, *n* (%)	2,551 (56.70)	1,223 (60.37)	776 (54.76)	552 (52.27)	<0.001***
Daily fruit intake, *n* (%)	1,717 (38.16)	828 (40.87)	535 (37.76)	354 (33.52)	0.001**
Alcohol consumption, *n* (%)					<0.001***
Never	3,619 (80.44)	1,572 (77.59)	1,194 (84.26)	853 (80.78)	
Heavy drinking	720 (16.0)	368 (18.16)	187 (13.20)	165 (15.62)	
Light/moderate drinking	160 (3.56)	86 (4.25)	36 (2.54)	38 (3.60)	
Family history of CHD, *n* (%)	163 (3.62)	83 (4.10)	48 (3.39)	32 (3.03)	0.006**
Family history of hypertension, *n* (%)	1,527 (33.94)	720 (35.53)	488 (34.44)	319 (30.21)	0.002**
Family history of diabetes, *n* (%)	466 (10.36)	186 (9.18)	128 (9.03)	152 (14.39)	<0.001***
SBP (mmHg)	131.23 ± 14.47	128.88 ± 14.76	132.01 ± 13.76	134.68 ± 14.05	<0.001***
DBP (mmHg)	81.20 ± 8.88	81.05 ± 9.18	81.00 ± 8.57	81.75 ± 8.70	0.07
Height (cm	161.10 ± 7.95	161.90 ± 8.07	160.39 ± 7.62	160.52 ± 7.99	<0.001***
Weight (kg)	63.50 ± 9.95	63.6 ± 9.95	62.83 ± 9.84	64.22 ± 10.06	0.002**
Waist circumference (WC) (cm)	80.32 ± 9.08	80.32 ± 8.84	79. 66 ± 9.37	81.20 ± 9.08	<0.001***
Body mass index (BMI) (kg/m^2^)	24.46 ± 3.35	24.25 ± 3.21	24.40 ± 3.29	24.93 ± 3.66	<0.001***
FBG (mmol/L)	5.31 ± 1.22	4.51 ± 0.35	5.29 ± 0.20	6.89 ± 1.57	<0.001***
Hemoglobin A1c (%)	5.52 ± 0.94	5.24 ± 0.56	5.38 ± 0.62	6.28 ± 1.38	<0.001***
Triglyceride (TG) (mmol/L)	1.55 ± 0.96	1.46 ± 0.88	1.55 ± 0.97	1.73 ± 1.06	<0.001***
Total cholesterol (TC) (mmol/L	4.71 ± 1.09	4.63 ± 1.04	4.76 ± 1.09	4.82 ± 1.18	<0.001***
HDL-C (mmol/L)	1.36 ± 0.38	1.37 ± 0.38	1.37 ± 0.38	1.32 ± 0.37	0.001**
LDL-C (mmol/L)	2.14 ± 1.00	2.10 ± 0.94	2.18 ± 1.01	2.19 ± 1.07	0.008**
Homocysteine (μmol/L)	9.68 ± 6.63	8.49 ± 5.71	9.93 ± 5.81	11.60 ± 8.53	<0.001***
VAI	2.07 ± 1.76	1.89 ± 1.62	2.08 ± 1.73	2.40 ± 2.01	<0.001***
TG/HDL-C	1.32 ± 1.12	1.22 ± 1.02	1.31 ± 1.10	1.52 ± 1.27	<0.001***
TyG	8.63 ± 0.57	8.42 ± 0.50	8.63 ± 0.52	8.99 ± 0.58	<0.001***
TyG-BMI	211.40 ± 35.05	204.68 ± 31.89	211.12 ± 33.70	224.89 ± 38.74	<0.001***
TyG-WC	693.96 ± 99.42	677.98 ± 92.88	689.05 ± 97.93	731.85 ± 103.87	<0.001***
TyG-WHtR	4.31 ± 0.63	4.19 ± 0.58	4.30 ± 0.62	4.57 ± 0.66	<0.001***

Daily vegetable intake: eats 300 g of vegetables a day. Daily fruit intake: eats 200 g of fruits a day.

LDL-C, low-density lipoprotein cholesterol; HDL-C, high-density lipoprotein cholesterol; CHD, coronary heart disease; SBP, systolic blood pressure; DBP, diastolic blood pressure; FBG, fasting blood glucose; TyG, triglyceride–glucose; WHtR, waist-to height ratio; VAI, visceral adiposity index.

*p< 0.05; **p< 0.01; ***p< 0.001.

### Demographics and baseline characteristics of the high-stroke-risk population

All the demographic and baseline characteristics were sorted by TyG quartiles and are shown in **Table 2**. Ultimately, a total of 1,038 high-stroke-risk participants were included. The mean age was 58.66 ± 9.77 years. Among the included participants, 55.88% have a sweet tooth, 89.98% had hypertension, 27.55% had DM, 2.89% had AF and/or VHD, 64.07% had dyslipidemia, 6.64% had a personal history of stroke, and 0.29% had a personal history of TIA. The smoking rate was 38.34%. In addition, 70.13% reported physical inactivity and 32.47% reported being overweight or obese. Moreover, 14.93% had a family history of stroke. Regarding the prevalence of cervical arterial atherosclerosis, 21.97% of the participants had increased IMT, 37.09% had carotid plaque, and 6.17% had CAS. The TyG-Q4 group showed a significantly increased proportion of men (*p* = 0.006) and was more likely to have higher SBP (*p*< 0.001), diastolic blood pressure (DBP) (*p*< 0.001), weight (*p*< 0.001), WC (*p*< 0.001), BMI (*p*< 0.001), VAI (*p*< 0.001), FBG (*p*< 0.001), hemoglobin A1c (*p*< 0.001), TG (*p*< 0.001), TC (*p*< 0.001), LDL-C (*p*< 0.001), and homocysteine (*p* = 0.047). In addition, the TyG-Q4 group had significantly higher values of the TyG parameters TyG-BMI, TyG-WC, and TyG-WHtR (all *p*< 0.001). The TyG-Q4 group also had a significantly higher proportion of carotid plaque (*p* = 0.021).

**Table 2:** Baseline characteristics by triglyceride–glucose (TyG) quartiles in the high-stroke-risk population.

	Total	Q1	Q2	Q3	Q4	*p*-value
TyG	–	≤8.570243	8.570243–8.946032	8.946032–9.369544	≥9.369544	–
*N*	1,038	260	260	259	259	–
Region						0.64
Urban, *n* (%)	656 (63.20)	164 (63.08)	160 (61.54)	172 (66.41)	160 (61.78)	
Rural, *n* (%)	382 (36.80)	96 (36.92)	100 (38.46)	87 (33.59)	99 (38.22)	
Age (years)	58.66 ± 9.77	59.11 ± 9.27	58.32 ± 10.30	58.61 ± 10.03	58.63 ± 9.50	0.83
Gender, male, *n* (%)	350 (33.72)	76 (29.23)	77 (29.62)	88 (33.98)	109 (42.08)	0.006**
Eating habits						0.72
High-salt diet	147 (14.16)	35 (13.46)	43 (16.54)	36 (13.90)	33 (12.74)	
Oil-heavy diet	311 (29.96)	76 (29.23)	84 (32.31)	73 (28.19)	78(30.12)	
Sweet tooth	580 (55.88)	149 (57.31)	133 (51.15)	150 (57.92)	148 (57.14)	
Meats and vegetables						0.36
Balanced	89 (8.57)	20 (7.69)	27 (10.38)	18 (6.95)	24 (9.27)	
Carnivorous diet	240 (23.12)	54 (20.77)	65 (25.00)	54 (20.85)	67 (25.88)	
Plant-based diet	709 (68.30)	186 (71.54)	168 (64.62)	187 (72.20)	168 (64.86)	
Daily vegetable intake, *n* (%)	543 (52.31)	136 (52.31)	135 (51.92)	135 (52.12)	137 (52.90)	0.99
Daily fruit intake, *n* (%)	347 (33.43)	87 (33.46)	93 (35.77)	81 (31.27)	86 (33.20)	0.78
Alcohol consumption, *n* (%)						0.88
Never	837 (80.64)	217 (83.46)	210 (80.77)	207 (7.92)	203 (78.38)	
Heavy drinking	164 (15.80)	35 (13.46)	40 (15.38)	43 (16.60)	46 (17.76)	
Light/moderate drinking	37 (3.56)	8 (3.08)	10 (3.85)	9 (3.47)	10 (3.85)	
Family history of CHD, *n* (%)	32 (3.08)	11 (4.23)	11 (4.23)	4 (1.54)	6 (2.32)	0.11
Family history of hypertension, *n* (%)	314 (30.25)	69 (26.54)	69 (26.54)	79 (30.50)	97 (37.45)	0.01*
Family history of diabetes, *n* (%)	151 (14.54)	25 (9.62)	32 (12.31)	44 (17.00)	50 (19.31)	0.004**
SBP (mmHg)	134.80 ± 14.06	132.48 ± 14.23	133.30 ± 12.96	136.35 ± 13.95	137.09 ± 14.59	<0.001***
DBP (mmHg)	81.79 ± 8.74	80.50 ± 8.29	80.69 ± 8.08	82.37 ± 9.32	83.63 ± 8.88	<0.001***
Height (cm)	160.54 ± 8.00	160.36 ± 0.15	160.30 ± 7.32	160.05 ± 7.80	161.46 ± 8.65	0.229
Weight (kg)	64.29 ± 10.08	60.44 ± 9.00	63.59 ± 9.62	65.44 ± 8.87	67.69 ± 11.27	<0.001***
Waist circumference (WC) (cm)	81.24 ± 9.12	77.58 ± 8.37	80.71 ± 8.25	83.02 ± 9.12	83.65 ± 9.49	<0.001***
BMI (kg/m^2^)	24.96 ± 3.67	23.55 ± 3.43	24.74 ± 3.26	25,61 ± 3.85	25.95 ± 3.66	<0.001***
FBG (mmol/L)	6.89 ± 1.58	6.26 ± 0.65	6.55 ± 0.89	6.92 ± 1.58	7.84 ± 2.21	<0.001***
Hemoglobin A1c (%)	6.33 ± 1.42	5.81 ± 0.98	6.06 ± 1.08	6.40 ± 1.25	7.05 ± 1.90	<0.001***
Triglyceride (TG) (mmol/L)	1.75 ± 1.09	0.97 ± 0.12	1.12 ± 0.32	1.80 ± 0.41	3.14 ± 1.22	<0.001***
Total cholesterol (TC) (mmol/L)	4.82 ± 1,21	4.54 ± 1.27	4.76 ± 1.13	4.88 ± 1.19	5.12 ± 1.20	<0.001***
HDL-C (mmol/L)	1.28 ± 0.47	1.56 ± 0.55	1.33 ± 0.48	1.18 ± 0.39	1.07 ± 0.27	<0.001***
LDL-C (mmol/L)	2.20 ± 1.08	2.05 ± 1.05	2.35 ± 1.03	2.30 ± 1.09	2.10 ± 1.11	0.002**
Homocysteine (μmol/L)	11.64 ± 8.59	12.69 ± 10.01	11.37 ± 8.33	10.63 ± 7.01	11.89 ± 8.64	0.047*
VAI	2.41 ± 2.02	0.91 ± 0.36	1.59 ± 0.57	2.42 ± 0.91	4.71 ± 2.60	<0.001***
TG/HDL-C	1.53 ± 1.27	0.56 ± 0.19	0.98 ± 0.30	1.51 ± 0.49	3.01 ± 1.60	<0.001***
TyG-BMI	224.89 ± 38.74	195.36 ± 28.55	216.75 ± 28.70	234.25 ± 35.19	253.33 ± 36.09	<0.001***
TyG-WC	731.85 ± 103.87	644.29 ± 72.61	707.42 ± 72.93	758.77 ± 83.77	817.37 ± 96.53	<0.001***
TyG-WHtR	4.57 ± 0.66	4.03 ± 0.48	4.42 ± 0.46	4.75 ± 0.57	5.07 ± 0.61	<0.001***
Increased IMT (%)	228 (21.97)	58 (22.31)	57 (21.92)	50 (19.31)	63 (24.32)	0.59
Carotid plaque (%)	385 (37.09)	80 (30.77)	92 (35.38)	100 (38.61)	113 (43.63)	0.02*
CAS (%)	64 (6.17)	12 (4.62)	12 (4.62)	18 (6.95)	22 (8.49)	0.18

LDL-C, low-density lipoprotein cholesterol; HDL-C, high-density lipoprotein cholesterol; CHD, coronary heart disease; SBP, systolic blood pressure; DBP, diastolic blood pressure; FBG, fasting blood glucose; TyG, triglyceride–glucose; WHtR, waist-to height ratio; VAI, visceral adiposity index.

*p< 0.05; **p< 0.01; ***p< 0.001.

### Relationship between TyG and cervical arterial atherosclerosis

**Table 3** shows the multivariate logistic analyses between TyG quartiles and carotid plaque in different models. The omnibus tests of model coefficients are shown in **Supplementary Table S2**. Compared with those in the TyG-Q1 group, participants in the TyG-Q2, TyG-Q3, and TyG-Q4 groups were more likely to have carotid plaque (model 4: OR = 1.85, 95%CI = 1.28–2.67; OR = 1.51, 95%CI = 1.05–2.18; OR = 1.29, 95%CI = 0.90–1.84; *p* = 0.01). The TyG-Q2 group had a significant increased risk of cervical atherosclerosis in all models, while no significant statistical difference was found for the TyG-Q4 group. After adjustment for age, sex, smoking, drinking, physical inactivity, hypertension, DM, AF and/or VHD, dyslipidemia, personal history of stroke/TIA, and family history of stroke, TyG was also found to be independent of all covariates. Logistic analysis between the TyG quartiles and increased IMT/CAS showed no statistical difference. In addition, ROC analysis was performed to examine the accuracy of the prediction of cervical artery atherosclerosis. As displayed in **Supplementary Figure S1**, TyG showed a significantly high area under the ROC curve (AUC) of 0.62 with a permutation *p*< 0.001.

**Table 3:** Odds ratios (95% confidence intervals) for triglyceride–glucose (TyG) and carotid plaque in the high-stroke-risk population.

TyG quartiles					
Model	Q1	Q2	Q3	Q4	*p*-value
1	Reference	1.74 (1.22–2.50)*	1.41 (0.99–2.01)	1.23(0.87–1.75)	0.021*
2	Reference	1.77 (1.23–2.54)*	1.42 (1.00–2.04)*	1.24 (0.87–1.76)	0.017*
3	Reference	1.79 (1.24–2.58)*	1.41 (0.99–2.02)	1.23 (0.86–1.76)	0.016*
4	Reference	1.85 (1.28–2.67)*	1.51 (1.05–2.18)*	1.29 (0.90–1.84)	0.010*
5	Reference	1.99 (1.28–3.09)*	1.51 (1.02–2.25)*	1.25 (0.87–1.81)	0.021*

Model 1: crude model; model 2: adjusted for age and sex; model 3: adjusted for age, sex, smoking, drinking, and physical inactivity; model 4: adjusted for age, sex, smoking, drinking, physical inactivity, hypertension, diabetes mellitus, atrial fibrillation/valvular heart disease, dyslipidemia, personal history of stroke/transient ischemic attack (TIA), and family history of stroke; model 5: adjusted for gender, family history of hypertension, family history of diabetes, systolic blood pressure (SBP), diastolic blood pressure (DBP), weight, waist circumference, body mass index (BMI), fasting blood glucose (FBG), hemoglobin A1c, triglyceride, total cholesterol, high-density lipoprotein cholesterol (HDL-C), low-density lipoprotein cholesterol (LDL-C), and homocysteine. *p<0.05.

### Relationship between TyG and carotid plaque in subgroup analysis

We further performed subgroup analysis using several daily habit and body fat indices. For the participants with DM, those in the TyG-Q2, TyG-Q3, and TyG-Q4 groups were more likely to have carotid plaque than those in TyG-Q1 (OR = 3.21, 95%CI = 1.52–6.80; OR = 2.20, 95%CI = 1.10–4.40; OR = 1.69, 95%CI = 0.84–3.39; *p* = 0.018) (**Table 4**). For participants in the non-overweight or obese group, those in TyG-Q2, TyG-Q3, and TyG-Q4 were more likely to have carotid plaque than those in TyG-Q1 (OR = 2.03, 95%CI = 1.28–3.20; OR = 1.56, 95%CI = 1.01–2.42; OR = 1.65, 95%CI = 1.05–2.58; *p* = 0.02). For subjects in the non-sweet tooth group, those in TyG-Q2, TyG-Q3, and TyG-Q4 were more likely to have carotid plaque than those in TyG-Q1 (OR = 2.60, 95%CI = 1.43–4.71; OR = 1.93, 95%CI = 1.11–3.36; OR = 1.38, 95%CI = 0.79–2.42; *p* = 0.01).

**Table 4:** Odds ratios (95% confidence intervals) of the subgroup analysis for triglyceride–glucose (TyG) and carotid plaque in the high-stroke-risk population.

Groups	TyG quartiles				*p-*value
	Q1	Q2	Q3	Q4	
Female	Reference	1.78 (1.12–2.81)	1.40 (0.89–2.19)	1.22 (077–1.92)	0.09
Male	Reference	2.03 (1.07–3.88)	1.80 (0.96–3.41)	1.38 (0.76–2.49)	0.12
DM	Reference	3.21 (1.52–6.80)*	2.20 (1.10–4.40)*	1.69 (0.84–3.39)	0.02
Non-DM	Reference	1.51 (0.98–2.32)	1.31 (0.84–2.02)	1.20 (0.78–1.84)	0.30
Overweight or obese	Reference	1.56 (0.82–2.96)	1.53 (0.77–3.03)	0.89 (0.47–1.69)	0.22
Non-overweight or obese	Reference	2.03 (1.28–3.20)**	01.56 (1.01–2.42)*	1.65 (1.05–2.58)*	0.02
Sweet tooth	Reference	1.48 (0.92–2.39)	1.23 (0.75–2.02)	1.22 (0.76–1.96)	0.46
Non-sweet tooth	Reference	2.60 (1.43–4.71)**	1.93 (1.11–3.36)*	1.38 (0.79–2.42)	0.01
Physical inactivity	Reference	1.67 (1.07–2.60)	1.54 (0.99–2.39)	1.33 (0.85–2.07)	0.12
Non-physical inactivity	Reference	2.28 (1.15–4.52)	1.33 (0.68–2.61)	1.16 (0.61–2.20)	0.11

Adjusted for age, sex, smoking, drinking, physical inactivity, hypertension, diabetes mellitus, atrial fibrillation/valvular heart disease, dyslipidemia, personal history of stroke/transient ischemic attack (TIA), and family history of stroke.

*p< 0.05; **p< 0.01.

## Discussion

In the current study, we found that the prevalence of a high stroke risk in the general population older than 40 years in eastern China was 23.47%. The TyG index in this population was higher than that in the low-stroke-risk population. Furthermore, it was found that the TyG index is an independent risk factor for carotid plaques in the high-stroke-risk population. Cervical arterial atherosclerosis is a notoriously pathophysiological process of ischemic stroke. These results suggest that the TyG index, a simple measure reflecting carotid plaques, is potentially useful in the early screening of individuals at high risk of stroke.

China faces the greatest challenge from stroke in the world (1). Screening of the risk factors of stroke was implemented in eastern China to improve management of the high-stroke-risk population. In the current study, a large percentage of the high-risk population emphasized the importance of stroke screening. The main contributors to stroke include behavioral risk factors (e.g., eating habits, exercise habits, smoking, and alcohol use) and preexisting conditions (hypertension, DM, dyslipidemia, and AF). All these factors may contribute to the regional high prevalence of cardiovascular disease (CVD) and cervical artery atherosclerosis.

Recently, several general population-based studies have investigated the correlation between TyG and cervical arterial atherosclerosis. The relationship between these two has been proven in postmenopausal women. The study compared the association between the structural and functional indices of subclinical atherosclerosis [i.e., carotid artery IMT, flow-mediated dilation of the brachial artery, and pulse wave velocity (PWV)] and the TyG index, separately for lean and overweight/obese women. The results showed that the TyG index is associated with carotid atherosclerosis and arterial stiffness mainly in lean postmenopausal women. The TyG index may serve as a useful marker for the identification of high-risk women in the normal-weight postmenopausal population (8). High blood pressure is a major cause of atherosclerosis that leads to stroke and myocardial infarction. Data from 77 hypertensive and 199 normotensive individuals were analyzed in a case–control study in Taiwan. The TyG index, IMT, and plaque presence were higher in hypertensive individuals compared to the control group. The TyG index was also significantly correlated with the carotid plaque score and the IMT of the CCA, ICA, and external carotid artery (ECA). Researchers have suggested that the TyG index is significantly associated with the carotid IMT, but could only predict early-stage subclinical atherosclerosis independent of hypertension history, age, sex, and BMI (9).

Other studies have focused on the TyG index and atherosclerotic CVD (ASCVD) (10, 11, 13, 15, 21–26). A retrospective observational cohort study addressed TyG and ASCVD in a large-scale population dataset (5,593,134 participants) older than 40 years. During 8.2 years of mean follow-up, a high TyG index was found to be associated with a significantly increased risk of future ASCVD events, including stroke, myocardial infarction, and both stroke and myocardial infarction. These studies showed that a high TyG index could be a significant predictor of future cardiovascular events. One study applied intravascular optical coherence tomography (OCT) to investigate the prognostic value of the TyG index combined with the morphological characteristics of vulnerable culprit coronary plaques in predicting cardiovascular outcomes. OCT, a cross-sectional and high-resolution intravascular imaging technique, allows the acquisition of detailed *in vivo* images of coronary plaque morphology characteristics, including plaque rupture (PR) and plaque erosion (PE). Researchers have found that the TyG index, combined with plaque characteristics, is a novel biomarker for cardiovascular outcomes (27).

In the present study, we investigated the association between the TyG index and carotid plaque in a high-stroke-risk population in China. The results showed that TyG is independently associated with carotid plaques in individuals at high risk of stroke. After adjusting for several established risk factors, subjects with higher TyG were still more likely to have carotid plaque. The TyG-Q2 group showed a significantly increased risk of cervical atherosclerosis, but the statistical difference was not significant for the TyG-Q4 group. Further studies may require bigger sample sizes and more rigorous statistical methods. In the subgroup analysis, the statistical differences were more significant for individuals with DM, those who are non-overweight or obese, and for those without a sweet tooth. We also found a significant effect of gender on TyG and of DM on carotid plaque. However, logistic analysis did not show much statistical difference between TyG and IMT or CAS. Compared with theTyG-Q1 group, subjects in the TyG-Q2, TyG-Q3, and TyG-Q4 groups were all more likely to have CAS, but showed no statistics difference. Our study also specifically confirmed the correlation between TyG and cervical arterial atherosclerosis. As previously mentioned, the measurement of TyG is inexpensive and readily available, and it is suitable for use as a screening indicator for the general population. It is easier to promote the use of this method in the general population as not all community hospitals have ultrasound equipment. Therefore, TyG is more advantageous compared to traditional parameters.

Previous general population-based studies also investigated the correlation between TyG or its related parameters and stroke. Several studies have focused on the value of TyG to estimate the risk of ischemic stroke (12, 13, 28). An 11-year follow-up study showed that elevated levels at both baseline and long-term updated cumulative average TyG index can independently predict ischemic stroke, but not intracerebral hemorrhage, in the general population (28). Du et al. estimated the prevalence of ischemic stroke from TyG-BMI in two general populations in Liaoning, northeast China. They discovered the potential usefulness of TyG-BMI to improve the risk stratification of ischemic stroke (14). Other studies found that the TyG index could predict the clinical outcomes of stroke, including poor outcomes after reperfusion therapy (17), an increased risk of stroke recurrence (16, 29), all-cause mortality (15, 16), and neurological worsening in patients with ischemic stroke (16, 18). As previously mentioned, most of the relevant studies in China have focused on the population of northeast area. Moreover, few studies have focused on the TyG index and the carotid ultrasound indices in stroke.

Our study, in accordance with other studies, showed that the TyG index is significantly higher in the high-stroke-risk population. In this study, we found that the prevalence rates of increased IMT, carotid plaque, and CAS in the high-stroke-risk general population older than 40 years were 21.97%, 39.3%, and 6.1%, respectively. We also discovered that the TyG index can be an independent predictor of carotid plaque in the high-stroke-risk population, which is a marker of cervical arterial atherosclerosis. However, we did not find a correlation between TyG and an increased IMT or CAS. A possible reason may be the relatively lower prevalence of CAS (6.1%) in this general population. Furthermore, a number of non-CAS participants still had accompanying carotid plaque when they were compared with the CAS group; as a result, some differences may have been obscured. Long-term follow-up studies are needed to further confirm the relationship between TyG and an increased IMT or CAS. In conclusion, our study provided more reference for the application of TyG as a clinically useful marker in the identification of individuals at high risk of carotid plaques or even cervical arterial atherosclerosis.

Why would TyG be associated with cervical arterial atherosclerosis? It might be linked to IR, while the TyG index is a marker of IR. IR is a common pathological condition in which cells are impaired in their ability to respond to the hormone insulin. IR contributes to the development of atherosclerosis *via* multiple mechanisms. Firstly, IR facilitates the formation of atherosclerosis through increased systemic inflammation (30) and endothelial dysfunction (31, 32) and promotes vulnerable plaques. Secondly, IR promotes platelet adhesion, activation, and aggregation, leading to the occlusion of cerebral arteries (33). Thirdly, IR could modify and influence the role of the modifiable stroke risk factors and contribute to the occurrence of atherosclerosis (34, 35). The association between TyG and cervical arterial atherosclerosis based on population levels is still controversial. Analyzing the composition of plaques and careful consideration of the mechanisms of IR would help clarify the exact mechanisms by which TyG contributes to cervical arterial atherosclerosis.

The present study has several limitations. Firstly, its cross-sectional design only allowed assessment of the associations between TyG and carotid plaques rather than their causal links. We could not establish cause-and-effect relationships between the observed associations. In addition, this cross-sectional survey could not provide data on the dynamic changes of TyG. Long-term follow-up studies are needed to further explore the predictive value of TyG for the estimation of ischemic stroke. Secondly, the general population used in the study is from Suzhou City. Varied populations from other cities in eastern China should be enrolled in future research to further confirm these initial findings. Thirdly, several other factors are potentially related to carotid atherosclerosis, but the current study mainly focused on age, sex and the “8+2” stroke risk factors in the final logistic regression models. Future research should further confirm the contribution of the other factors.

In conclusion, the TyG index was significantly associated with carotid plaques and is a convenient, reliable, and practical for clinical use. This index could be a practical indicator for ultrasound-detected cervical artery atherosclerosis, especially for carotid plaques. Furthermore, the statistical differences were more significant for non-overweight or obese and non-sweet tooth individuals, implying that, even for individuals with normal anthropometric features, TyG could still present a potential risk of cervical arterial atherosclerosis. Primarily, TyG may serve as a simple and low-cost marker for the screening of high-stroke-risk groups who need further ultrasonography. It is beneficial to early primary stroke prevention.

## Data availability statement

The original contributions presented in the study are included in the article/**Supplementary Material**. Further inquiries can be directed to the corresponding authors.

## Ethics statement

This study was reviewed and approved by the Ethics Committee of the First Affiliated Hospital of Soochow University. Patients/participants provided written informed consent to participate in this study. Written informed consent was obtained from individual(s) for the publication of any potentially identifiable images or data included in this article.

## Author contributions

XT and LZ conceived the research and wrote the main manuscript text. YL and YZ participated in the recruitment of the sample population. XT, LZ, and YL acquired the data and analyzed the results. XC helped in the interpretation of the results and revised the manuscript. YY and QF guided the process, interpreted the results, and revised the manuscript. All authors contributed to the article and approved the submitted version.

## Funding

This work was supported by the National Key R&D Program of China (no. 2017YFC0114300 to QF); Shanghai Sailing Program (no. 17YF142600, to YY); Startup Foundation of School of Public Health (no. IDF201360/009, to YY); Fudan University (no. KBH2306002, to YY); the National Natural Science Foundation of China (no. 82001125, to XT); and the Natural Science Foundation of Jiangsu Province (no. BK20180201, to XT).

## Conflict of interest

The authors declare that the research was conducted in the absence of any commercial or financial relationships that could be construed as a potential conflict of interest.

## Publisher’s note

All claims expressed in this article are solely those of the authors and do not necessarily represent those of their affiliated organizations, or those of the publisher, the editors and the reviewers. Any product that may be evaluated in this article, or claim that may be made by its manufacturer, is not guaranteed or endorsed by the publisher.

## References

[1] Wang YJ Li ZX Gu HQ Zhai Y Jiang Y Zhao XQ. China Stroke statistics 2019: A report from the national center for healthcare quality management in neurological diseases, China national clinical research center for neurological diseases, the Chinese stroke association, national center for chronic and non-communicable disease control and prevention, Chinese center for disease control and prevention and institute for global neuroscience and stroke collaborations. Stroke Vasc Neurol (2020) 5:211–39. doi: 10.1136/svn-2020-000457 PMC7548521 32826385

[2] Qi W Ma J Guan T Zhao D Abu-Hanna A Schut M. Risk factors for incident stroke and its subtypes in China: A prospective study. J Am Heart Assoc (2020) 9:e016352. doi: 10.1161/JAHA.120.016352 33103569 PMC7763402

[3] Spence JD. China Stroke statistics 2019: a wealth of opportunities for stroke prevention. Stroke Vasc Neurol (2020) 5:240–1. doi: 10.1136/svn-2020-000529 PMC7548513 32826384

[4] Tadi P Lui F. Acute stroke. Treasure Island (FL: StatPearls (2021).

[5] Qaja E Tadi P Theetha Kariyanna P. Carotid artery stenosis. Treasure Island (FL: StatPearls (2021). 28723054

[6] Raimi TH Dele-Ojo BF Dada SA Fadare JO Ajayi DD Ajayi EA. Triglyceride-glucose index and related parameters predicted metabolic syndrome in nigerians. Metab syndr. relat. Disord (2021) 19:76–82. doi: 10.1089/met.2020.0092 33170086 PMC7929914

[7] Ahn N Baumeister SE Amann U Rathmann W Peters A Huth C. Visceral adiposity index (VAI), lipid accumulation product (LAP), and product of triglycerides and glucose (TyG) to discriminate prediabetes and diabetes. Sci Rep (2019) 9:9693. doi: 10.1038/s41598-019-46187-8 31273286 PMC6609728

[8] Lambrinoudaki I Kazani MV Armeni E Georgiopoulos G Tampakis K Rizos D. The TyG index as a marker of subclinical atherosclerosis and arterial stiffness in lean and overweight postmenopausal women. Heart Lung Circ (2018) 27:716–24. doi: 10.1016/j.hlc.2017.05.142 28690023

[9] Alizargar J Bai CH. Comparison of carotid ultrasound indices and the triglyceride glucose index in hypertensive and normotensive community-dwelling individuals: A case control study for evaluating atherosclerosis. Medicina (Kaunas) (2018) 54:71 doi: 10.3390/medicina54050071 PMC6262598 30344302

[10] Jin JL Cao YX Wu LG You XD Guo YL Wu NQ. Triglyceride glucose index for predicting cardiovascular outcomes in patients with coronary artery disease. J Thorac Dis (2018) 10:6137–46. doi: 10.21037/jtd.2018.10.79 PMC6297409 30622785

[11] Mao Q Zhou D Li Y Wang Y Xu SC Zhao XH. The triglyceride-glucose index predicts coronary artery disease severity and cardiovascular outcomes in patients with non-ST-Segment elevation acute coronary syndrome. Dis Markers (2019) 2019:6891537. doi: 10.1155/2019/6891537 31281548 PMC6594265

[12] Shi W Xing L Jing L Tian Y Yan H Sun Q. Value of triglyceride-glucose index for the estimation of ischemic stroke risk: Insights from a general population. Nutr. metab. Cardiovasc Dis NMCD (2020) 30:245–53. doi: 10.1016/j.numecd.2019.09.015 31744716

[13] Zhao Y Sun H Zhang W Xi Y Shi X Yang Y. Elevated triglyceride-glucose index predicts risk of incident ischaemic stroke: The rural Chinese cohort study. Diabetes Metab (2021) 47:101246. doi: 10.1016/j.diabet.2021.101246 33722769

[14] Du Z Xing L Lin M Sun Y. Estimate of prevalent ischemic stroke from triglyceride glucose-body mass index in the general population. BMC Cardiovasc Disord (2020) 20:483. doi: 10.1186/s12872-020-01768-8 33183220 PMC7663857

[15] Zhang B Liu L Ruan H Zhu Q Yu D Yang Y. Triglyceride-glucose index linked to hospital mortality in critically ill stroke: An observational multicentre study on eICU database. BMC Cardiovasc Disord (2020) 7:591036. doi: 10.3389/fmed.2020.591036 PMC7655911 33195355

[16] Zhou Y Pan Y Yan H Wang Y Li Z Zhao X. Triglyceride glucose index and prognosis of patients with ischemic stroke. Front Neurol (2020) 11:456. doi: 10.3389/fneur.2020.00456 32587566 PMC7297915

[17] Lee M Kim CH Kim Y Jang MU Mo HJ Lee SH. High triglyceride glucose index is associated with poor outcomes in ischemic stroke patients after reperfusion therapy. Cerebrovasc. Dis (Basel Switzerland) (2021) 50:691–9. doi: 10.1159/000516950 34229319

[18] Hou Z Pan Y Yang Y Yang X Xiang X Wang Y. An analysis of the potential relationship of triglyceride glucose and body mass index with stroke prognosis. Front Neurol (2021) 12:630140. doi: 10.3389/fneur.2021.630140 33967936 PMC8101495

[19] Yu Y Zhang FL Yan XL Zhang P Guo ZN Yang Y. Visceral adiposity index and cervical arterial atherosclerosis in northeast China: a population based cross-sectional survey. Eur J Neurol Off J Eur Fed Neurol. Soc. (2021) 28:161–71. doi: 10.1111/ene.14513 32896952

[20] Stroke screening and prevention project committee technical specification of stroke screening and prevention in China. Chin J Front Med Sci (2013) 9:44–50. doi: 10.3969/j.issn.1674-7372.2013.09.017

[21] Hong S Han K Park CY. The triglyceride glucose index is a simple and low-cost marker associated with atherosclerotic cardiovascular disease: a population-based study. BMC Med (2020) 18:361. doi: 10.1186/s12916-020-01824-2 33234146 PMC7687762

[22] Ding X Wang X Wu J Zhang M Cui M. Triglyceride-glucose index and the incidence of atherosclerotic cardiovascular diseases: a meta-analysis of cohort studies. Cardiovasc Diabetol (2021) 20:76. doi: 10.1186/s12933-021-01268-9 33812373 PMC8019501

[23] Wang A Tian X Zuo Y Chen S Meng X Wu S. Change in triglyceride-glucose index predicts the risk of cardiovascular disease in the general population: a prospective cohort study. Cardiovasc Diabetol (2021) 20:113. doi: 10.1186/s12933-021-01305-7 34039351 PMC8157734

[24] Si S Li J Li Y Li W Chen X Yuan T. Causal effect of the triglyceride-glucose index and the joint exposure of higher glucose and triglyceride with extensive cardio-cerebrovascular metabolic outcomes in the UK biobank: A mendelian randomization study. Front Cardiovasc Med (2020) 7:583473. doi: 10.3389/fcvm.2020.583473 33553250 PMC7863795

[25] Ma X Dong L Shao Q Cheng Y Lv S Sun Y. Triglyceride glucose index for predicting cardiovascular outcomes after percutaneous coronary intervention in patients with type 2 diabetes mellitus and acute coronary syndrome. Nutrients (2020) 19:31. doi: 10.1186/s12933-020-01006-7 PMC7063826 32156279

[26] Hu C Zhang J Liu J Liu Y Gao A Zhu Y. Discordance between the triglyceride glucose index and fasting plasma glucose or HbA1C in patients with acute coronary syndrome undergoing percutaneous coronary intervention predicts cardiovascular events: a cohort study from China. Cardiovasc Diabetol (2020) 19:116. doi: 10.1186/s12933-020-01091-8 32703284 PMC7379768

[27] Zhao X Wang Y Chen R Li J Zhou J Liu C. Triglyceride glucose index combined with plaque characteristics as a novel biomarker for cardiovascular outcomes after percutaneous coronary intervention in ST-elevated myocardial infarction patients: an intravascular optical coherence tomography study. Cardiovasc Diabetol (2021) 20:131. doi: 10.1186/s12933-021-01321-7 34183007 PMC8240222

[28] Wang A Wang G Liu Q Zuo Y Chen S Tao B. Triglyceride-glucose index and the risk of stroke and its subtypes in the general population: an 11-year follow-up. Cardiovasc Diabetol (2021) 20:46. doi: 10.1186/s12933-021-01238-1 33602208 PMC7893902

[29] Nam KW Kwon HM Lee YS. High triglyceride-glucose index is associated with early recurrent ischemic lesion in acute ischemic stroke. Cardiovasc Diabetol (2021) 11:15335. doi: 10.1038/s41598-021-94631-5 PMC8319389 34321520

[30] Matulewicz N Karczewska-Kupczewska M. Insulin resistance and chronic inflammation. Postepy Hig Med Dosw (Online) (2016) 70:1245–58. doi: 10.5604/17322693.1226662 28026827

[31] Bornfeldt KE Tabas I. Insulin resistance, hyperglycemia, and atherosclerosis. Cell Metab (2011) 14:575–85. doi: 10.1016/j.cmet.2011.07.015 PMC3217209 22055501

[32] Deng XL Liu Z Wang C Li Y Cai Z. Insulin resistance in ischemic stroke. Metab Brain Dis (2017) 32:1323–34. doi: 10.1007/s11011-017-0050-0 28634787

[33] Moore SF Williams CM Brown E Blair TA Harper MT Coward RJ. Loss of the insulin receptor in murine megakaryocytes/platelets causes thrombocytosis and alterations in IGF signalling. Cardiovasc Res (2015) 107:9–19. doi: 10.1093/cvr/cvv132 25902782 PMC4476412

[34] Maria Z Campolo AR Scherlag BJ Ritchey JW Lacombe VA. Dysregulation of insulin-sensitive glucose transporters during insulin resistance-induced atrial fibrillation. Biochim Biophys Acta Mol basis Dis (2018) 1864:987–96. doi: 10.1016/j.bbadis.2017.12.038 29291943

[35] Soleimani M. Insulin resistance and hypertension: new insights. Kidney Int (2015) 87:497–9. doi: 10.1038/ki.2014.392 25723632

