# Supplementary material for: Relationship between triglyceride–glucose index and carotid plaques in a high-stroke-risk population in southeast china: A population-based cross-sectional survey
Source: Front Endocrinol (Lausanne). 2022 Oct 12;13:1023867. doi: 10.3389/fendo.2022.1023867 (PMC9596760; doi:10.3389/fendo.2022.1023867)
Supplement: Supplementary file 1 [file DataSheet_1.docx]

**Supplementary Figure 1**

ROC curves generated for prediction of cervical artery atherosclerosis. Receiver operating characteristic (ROC) curve is generated for TyG (orange, AUC = 0.62, p < 0.001), TG (green, AUC = 0.57, p < 0.001) and FBG (purple, AUC = 0.52, p = 0.31) with cervical artery atherosclerosis, separately.


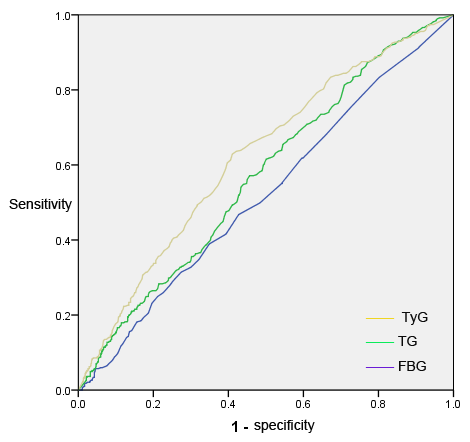


Supplementary Table 1. Interaction analysis of the subgroup analysis for TyG and carotid plaque in high-stroke-risk population

| Groups | TyG | | carotid plaque | | TyG * carotid plaque | |
| --- | --- | --- | --- | --- | --- | --- |
|  | F | *p* | F | *p* | F | *p* |
| Gender | 4.29 | 0.005 | 0.52 | 0.47 | 0.11 | 0.95 |
| DM (yes/no) | 1.51 | 0.21 | 12.69 | < 0.001 | 0.86 | 0.46 |
| Overweight or obese(yes/no) | 1.31 | 0.27 | 0.22 | 0.64 | 1.45 | 0.29 |
| a sweet tooth (yes/no) | 0.88 | 0.45 | 2.47 | 0.12 | 0.88 | 0.45 |
| Physical inactivity (yes/no) | 0.46 | 0.71 | 0.79 | 0.37 | 0.50 | 0.69 |

Supplementary Table 2. Omnibus tests of Model Coefficients

|  |  |  | Chi-square | df | Sig. |
| --- | --- | --- | --- | --- | --- |
| Model 1 | Step 1 | Step | 9.80 | 3 | 0.02 |
|  |  | Block | 9.80 | 3 | 0.02 |
|  |  | Model | 9.80 | 3 | 0.02 |
| Model 2 | Step 1 | Step | 11.04 | 5 | 0.04 |
|  |  | Block | 11.04 | 5 | 0.04 |
|  |  | Model | 11.04 | 5 | 0.04 |
| Model 3 | Step 1 | Step | 20.52 | 9 | 0.02 |
|  |  | Block | 20.52 | 9 | 0.02 |
|  |  | Model | 20.52 | 9 | 0.02 |
| Model 4 | Step 1 | Step | 47.89 | 12 | < 0.001 |
|  |  | Block | 47.89 | 12 | < 0.001 |
|  |  | Model | 47.89 | 12 | < 0.001 |
| Model 5 | Step 1 | Step | 40.00 | 16 | 0.001 |
|  |  | Block | 40.00 | 16 | 0.001 |
|  |  | Model | 40.00 | 16 | 0.001 |
